# Research hotspots and frontiers in post-stroke dysphagia: a bibliometric analysis study

**DOI:** 10.3389/fneur.2024.1343469

**Published:** 2024-02-02

**Authors:** Bilian Guo, Mengwei Liu, Zhiyong Wang, Zhipeng Yan

**Affiliations:** ^1^Department of Rehabilitation Medicine, First Affiliated Hospital of Fujian Medical University, Fuzhou, China; ^2^Department of Rehabilitation Medicine, National Regional Medical Center, Binhai Campus of the First Affiliated Hospital, Fujian Medical University, Fuzhou, China; ^3^Neuropsychiatric Prevention and Treatment Hospital of Fuzhou Second General Hospital, Fuzhou, China

**Keywords:** dysphagia, stroke, CiteSpace, visual analysis, bibliometric

## Abstract

**Background:**

Dysphagia is a common complication of stroke that can result in serious consequences. In recent years, more and more papers on post-stroke dysphagia have been published in various journals. However, there is still a lack of bibliometric analysis of post-stroke dysphagia. This study visually analyzes the global research situation of post-stroke dysphagia from 2013 to 2022, aiming to explore the current research status, frontier trends, and research hotspots in this field.

**Methods:**

Articles and reviews relevant to post-stroke dysphagia were obtained and retrieved from the Web of Science core collection database in the last 10 years (from 2013 to 2022). CiteSpace and Microsoft Excel 2019 were used for bibliographic analysis.

**Results:**

A total of 1,447 articles were included in the analysis. The number of publications showed an overall upward trend, from 72 in 2013 to 262 in 2022. The most influential authors, institutions, journals, and countries were Hamdy S, University of London, Dysphagia, and the People's Republic of China. An analysis of keywords and the literature indicated that current studies in the field of post-stroke dysphagia focused on dysphagia and aspiration, dysphagia classification, dysphagia rehabilitation, and daily living.

**Conclusion:**

This bibliometric analysis reveals the latest advancements and emerging trends in the field of post-stroke dysphagia, spanning the years 2013 to 2022. It highlights the paramount importance of conducting large-scale randomized controlled trials examining the efficacy of dysphagia screening protocols and non-invasive intervention techniques in improving the quality of life for these patients. Such research efforts hold significant academic implications for the development of evidence-based treatment strategies in this field.

## Introduction

Stroke is a group of cerebrovascular diseases caused by organic brain damage, with common clinical characteristics of sudden onset and rapid development of localized or diffuse brain function deficits (1, 2). Stroke is the main cause of disability and death (3). Due to the aging population, high blood pressure, and other risk factors, as well as poor management, the incidence rate of stroke continues to rise, which will further increase the emotional and economic burden on families and society (4–6). Swallowing is one of the most basic physiological activities that humans rely on for survival (7). Depending on the part of the food passing through, it can be generally divided into oral, pharyngeal, and esophageal stages. Any structural or functional damage in any of these stages can lead to the occurrence of dysphagia (8–10). Dysphagia is a common complication of stroke, and relevant data show that 28 to 67% of stroke patients have dysphagia (11).

The diagnosis of post-stroke dysphagia is relatively easy, but determining the location and nature of dysphagia requires detailed clinical evaluation and instrumental examination (12). Clinical evaluation includes dysphagia screening or a comprehensive evaluation of the orofacial structure and function by a speech and language pathologist. Instrumental evaluation includes the Video Fluoroscopic Swallowing Study (VFSS) and Flexible Endoscopic Evaluation of Swallowing (FEES). Post-stroke dysphagia can cause an inability to eat normally and can lead to serious consequences such as malnutrition, aspiration pneumonia, and psychological disorders, resulting in prolonged hospitalization and increased complications (13–15). Treatment for post-stroke dysphagia includes dietary interventions, behavioral interventions, nutritional interventions, interventions to improve oral health, pharmacological treatment, and neurostimulation treatment (16). For instance, research has shown that nutrients given through the gastrointestinal tract are more easily absorbed and help maintain the integrity of the intestinal mucosal structure and intestinal barrier. Therefore, enteral nutrition such as nasogastric tubes and percutaneous endoscopic gastrostomy (PEG) are of great significance for patients with post-stroke dysphagia (17). Although there is increasing consensus on the effectiveness of existing treatment methods in promoting the rehabilitation of post-stroke dysphagia, there are still many problems and uncertainties that need to be explored. Therefore, research on post-stroke dysphagia is necessary and valuable. In recent years, more and more papers on post-stroke dysphagia have been published in various journals (18–21). However, there is still a lack of scientific and measurement analysis of post-stroke dysphagia.

Bibliometric analysis uses quantitative methods such as mathematics and statistics to describe, evaluate, and monitor research in a specific field in order to reveal the research structure and trends in a certain discipline (22, 23). In recent years, using CiteSpace for literature and metrological analysis has become a research hotspot for scholars at home and abroad. CiteSpace is a literature metrological modeling software that can be used for basic literature analysis, such as citation analysis, international and institutional cooperation analysis, author cooperation analysis, dual-map overlay of journals, keywords analysis, and clustering and mutation analysis. Through analysis, it provides insights into the structure, social network, and topic interests of the field (24, 25).

Hence, this study conducted a comprehensive review of publications in the field of post-stroke dysphagia research and used CiteSpace for bibliometric analysis. Based on the analysis results, we could help researchers quickly understand the main progress, research hotspots, and frontiers of post-stroke swallowing dysfunction, addressing the lack of quantitative analysis in this field.

## Materials and methods

### Data source and search strategy

The Web of Science database encompasses a diverse range of disciplines, with its fundamental principles encompassing data structuring, subject categorization, citation analysis, an international perspective, and scalability. It is the preeminent database utilized for bibliometric analysis (26). Earlier studies have convincingly established the efficacy of bibliometric analysis conducted on the WoSCC database (27, 28). The literature data relevant to the topic of this bibliometric study were obtained and retrieved from the core collection database in the Web of Science (WoS). Searches were conducted using the following MeSH terms: (((swallowing) OR (dysphagia)) OR (swallowing disorder)) AND (stroke). Our literature search was limited to the time period between 1 January 2013 and 31 December 2022, with a yearly time slice. This approach aims to prevent potential information obsolescence and degradation in data quality associated with long-term datasets, ensuring the timeliness and reliability of the retrieved information. Additionally, limiting the search duration to a decade enhances the efficiency and feasibility of data analysis and visualization, ultimately reducing the complexity and cost associated with data processing.

### Inclusion and exclusion criteria

**Figure 1:**
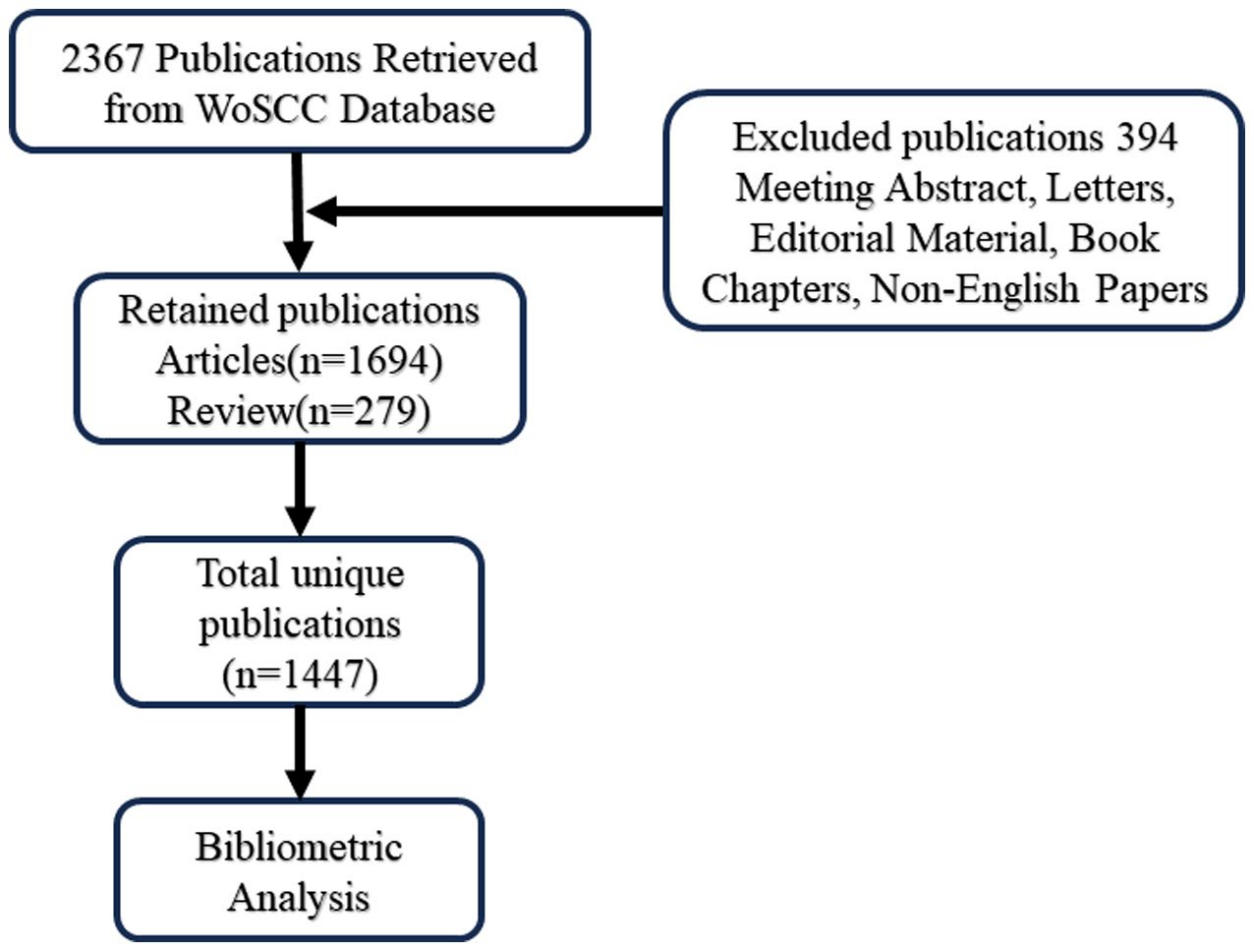
Flowchart of literature inclusion.

After a thorough examination of the article titles and abstracts, only those related to post-stroke dysphagia were selected for this bibliometric analysis. Other document types, such as meeting abstracts, letters, editorial materials, and book chapters, were excluded. Publications written in English were the only ones considered for this analysis. Figure 1 shows the flow chart of the selection process. In the end, a total of 1,447 records were deemed suitable and included in the final bibliometric analysis.

### Software parameter settings

CiteSpace V (version 6.2.R4; Drexel University, United States) and Microsoft Excel 2019 were used for bibliographic analysis, with the “Time Sliding” value set to 1 year and the type of node selected based on the analysis’s purpose. In this study, we employed “country, institution, author, reference, keyword” as node types for visual analysis, and only one node type can be selected at a time.

### Interpretation of main parameters

#### Node circle and the link between nodes

The size of a node circle in the countries or institutional co-authorship network represents the number of papers published as well as the frequency of authors in the co-occurrence network. Meanwhile, a link between nodes shows the presence of a co-authorship or co-occurrence relationship.

#### Betweenness centrality

The index of betweenness centrality is an indicator of the importance of nodes in a network.

#### Dual-map overlaps

The dual-map overlaps of journals are a novel method of visualizing the distribution of articles and citation trajectories across different fields. It provides insight into interdisciplinary relationships in the academic world. The citation line, represented by a curve, presents the context of the citation in a comprehensive manner. The use of the Z-score and F-score allowed for re-adjustment and standardization of the citation data, enabling the identification of major citation paths on the dual-map.

#### Burst detection

The purpose of burst detection is to identify significant increases in citation numbers within a particular timeframe.

#### H-index

H-index is a metric used to evaluate academic success. It is calculated by counting the number of publications that have been cited more than H times.

## Results

### Publication analysis

In this study, a total of 1,447 articles were included in Supplementary Figure S1 displays the annual publication and citation distribution of literature related to post-stroke dysphagia. We identified the overall and prominent sub-topic (clinical neurology) trend lines, represented by orange and blue colors, respectively. The orange line represents the general trend observed in a broader field, whereas the blue line highlights the specific trend in clinical neurology. As shown in Supplementary Figure S1A, it is evident that the number of publications in this field has been increasing, climbing from 72 (4.98%) in 2013 to 262 (18.11%) in 2022. Linear regression analysis indicates that the time trend of the number of publications in the past decade was significantly correlated (*R*^2^ = 0.9466). However, it should be noted that the growth rate of clinical neurology was relatively slow. Supplementary Figure S1B demonstrates the distribution of citation frequency, indicating that the number of citations has been increasing year by year, and the growth of clinical neurology aligned with the overall growth pattern. By utilizing an exponential growth model to evaluate the correlation between the number of citations and the publication year, it was found that the model aligns with the observed trend.

Supplementary Figure S2 shows that 2022 recorded the most published papers (*n* = 262) and open access papers (*n* = 189). The highest number of citations per paper was 37.16 in 2016. The largest number of citations (*n* = 3,456) and the H index (*n* = 31) also occurred in 2016.

### Authoritative journals analysis

A total of 1,447 papers on post-stroke dysphagia were published in 476 academic journals in this study. Table 1 summarizes the list of the top 15 academic journals, ranked by the number of publications. In the top 15 journals, dysphagia contributed the highest number of published articles (*n* = 95), and the greatest H-index value (*n* = 23), followed by the Journal of Stroke and Cerebrovascular Diseases (*n* = 75). Medicine and Stroke were tied for third place with 35 papers each. The International Journal of Stroke presented with the highest impact factor of 6.7 in 2022 and the largest quantity of citations per paper. The academic journal Frontiers in Neurology had the most open access (*n* = 34).

**Table 1:** Top 15 paper journals based on the number of publications.

Journal	Publications	Citations	Citation per paper	Open access	WoS categories	IF(2022)	Quartile	H-index
Dysphagia	99	1950	19.7	29	Otorhinolaryngology	2.6	Q2	23
Journal of Stroke and Cerebrovascular Diseases	75	954	12.72	16	Neurosciences; Peripheral Vascular Disease	2.5	Q3; Q3	17
Medicine	35	209	5.97	35	Medicine; General and Internal	1.6	Q3; Q3	8
Stroke	35	1,307	37.34	33	Clinical Neurology; Peripheral Vascular Disease	8.3	Q1; Q1	19
Frontiers in Neurology	34	245	7.21	34	Clinical Neurology; Neurosciences	3.4	Q2; Q2	9
Archives of Physical Medicine and Rehabilitation	21	329	15.67	4	Rehabilitation; Sport Sciences	4.3	Q1; Q1	11
Journal of Oral Rehabilitation	21	553	26.33	2	Dentistry, Oral Surgery & Medicine	2.9	Q2	13
Neurogastroenterology and Motility	20	461	23.05	6	Gastroenterology & Hepatology; Clinical Neurology; Neurosciences	3.5	Q2; Q3; Q2	12
Neurorehabilitation	18	188	10.44	2	Clinical Neurology; Rehabilitation	2	Q4; Q2	8
Annals of Rehabilitation Medicine	16	100	6.25	16	Rehabilitation	1.3	Q3	7
PLOS ONE	16	438	27.38	16	Multidisciplinary Sciences	3.7	Q2	10
BMC Neurology	15	154	10.27	15	Clinical Neurology	2.6	Q2	7
Cerebrovascular Diseases	15	375	25	7	Clinical Neurology; Peripheral Vascular Disease	2.9	Q3; Q3	10
European Journal of Neurology	15	337	22.47	3	Clinical Neurology; Neurosciences	5.1	Q1; Q2	10
International Journal of Stroke	15	842	56.13	5	Clinical Neurology; Peripheral Vascular Disease	6.7	Q1; Q1	9

WoS, Web of Science; IF, Impact Factor; JCR, Journal Citation Reports.

**Figure 2:**
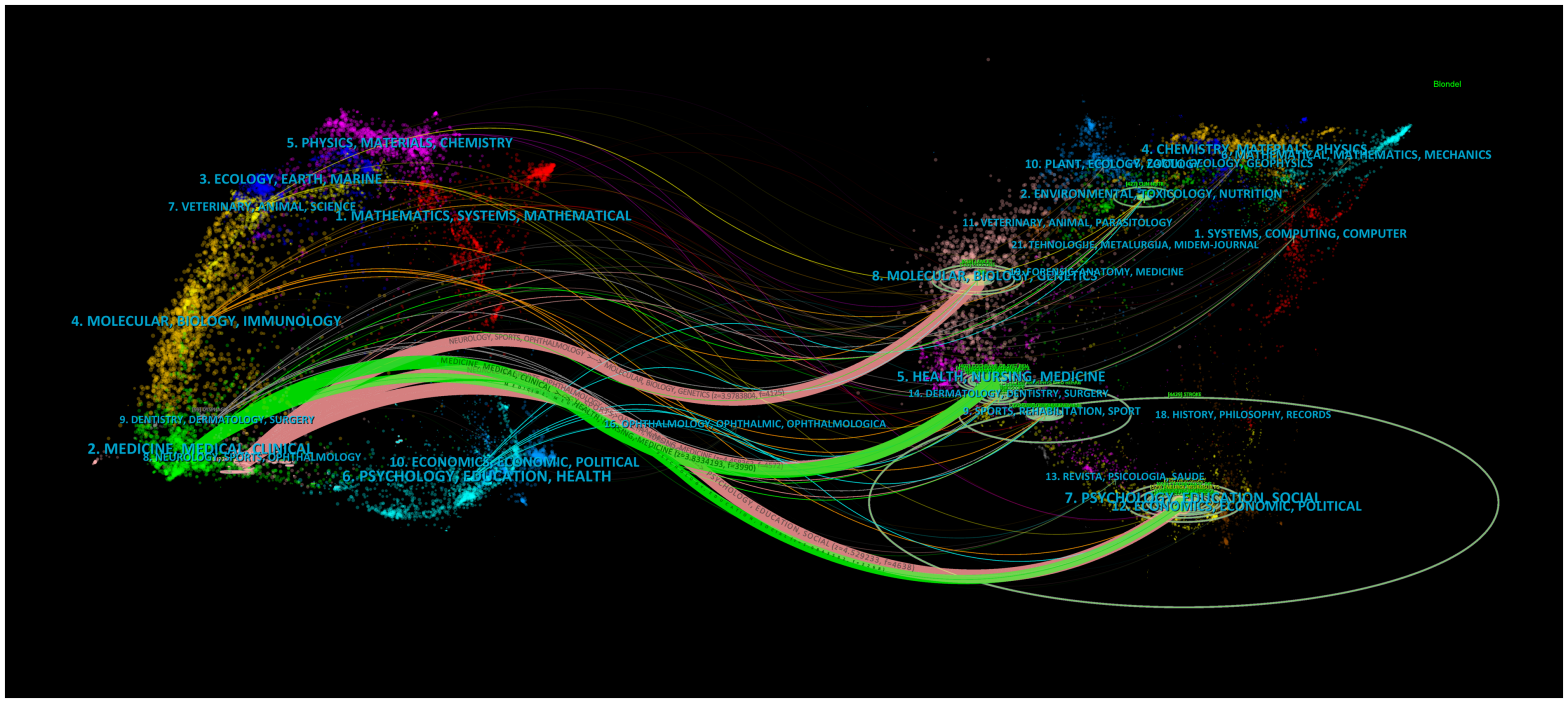
Dual-map overlaps of journals in post-stroke dysphagia. The map is assigned to two parts, with the citing journals listed on the left and the cited journals on the right.

Dual-map overlaps of journals are shown in Figure 2. The map was assigned to two parts, with the citing journals listed on the left and the cited journals on the right. The Z-score function was used to highlight a more fluid trajectory, with higher scores represented by thicker lines. Five major citation trajectories were determined (pink and green), with journals in Medicine, Neurology, Sports, and Ophthalmology (pink trajectory) being more frequently cited by Psychology, Education, Social (*Z* = 4.53, *f* = 4,638), Health, Nursing, Medicine (*Z* = 4.46, *f* = 4,572), and Molecular, Biology, and Genetics (*Z* = 3.98, *f* = 4,125) fields. Additionally, journals in Medicine, Medical, and Clinical (green track) were influenced by journals in Health, Medicine, Medical (*Z* = 3.83, *f* = 3,990), and Psychology, Education, and Social (*Z* = 1.98, *f* = 2,268).

### Subject category analysis

The 1,447 articles included in our study were classified into a total of 81 WoS subject categories. Supplementary Figure S3 shows the top 15 published disciplines ranked by the number of publications. Clinical neurology had the largest number of papers (*n* = 338), citations (*n* = 6,814), open-access papers (*n* = 195), and H-index value (*n* = 43). Peripheral vascular disease had the largest average number of citations per article (*n* = 24.26), followed by clinical neurology (*n* = 20.16) and nutrition dietetics (*n* = 18.62).

### Authoritative countries, institutions, and authors analysis

A total of 84 countries contributed to the publication of research on post-stroke dysphagia. Supplementary Figure S4A shows the top 15 countries based on the number of publications, with China having the highest number of publications (*n* = 182) and open access papers (*n* = 235). The USA had the highest H-index (*n* = 37) and the most citations (*n* = 4,871). Switzerland had the highest number of citations per paper (*n* = 48.12), followed by Spain (*n* = 29.28) and England (*n* = 24.75). Supplementary Figure S4B shows the top 11 countries with the strongest citation bursts. Sweden had the highest burst strength, with a score of 4.61 from 2015 to 2018, indicating a significant focus on post-stroke dysphagia research in Sweden during this period.

Supplementary Figure S5A displays information on the top 15 institutions based on the number of research papers published. The University of London ranked first with 49 publications, followed by the University of Manchester with 44 and Harvard University with 37 publications. The University of Munster had the highest citation rate of 39.64 per paper. When analyzing the burstness of institutions in Supplementary Figure S5B, it was observed that the Veterans Health Administration (VHA) and the US Department of Veterans Affairs were tied for first place, both scoring 5 from 2013 to 2014. Dongseo University ranked third with a burst strength score of 4.07 from 2019 to 2020.

Supplementary Figure S6A summarizes the top 15 most authoritative authors. Hamdy S had the largest number of papers (*n* = 25), with 22 of them being open access. Dziewas R had the highest number of citations (*n* = 915) and also shared the highest H-index (*n* = 15) with Clave P and Hamdy S. Additionally, Dziewas R had the greatest number of citations per paper, with a value of 45.75. Supplementary Figure S6B displays the top 15 authors with the strongest citation bursts. Park Ji-Su had the highest burst strength, scoring 4.12 from 2019 to 2020. Clave P had a score of 4.12 in burst strength, followed by Yoshimura Y with a score of 3.25 from 2020 to 2022. These scores indicated that both authors had a keen interest in the study of post-stroke dysphagia during this period.

### Coauthorship analysis of countries, institutions, and authors analysis

**Figure 3:**
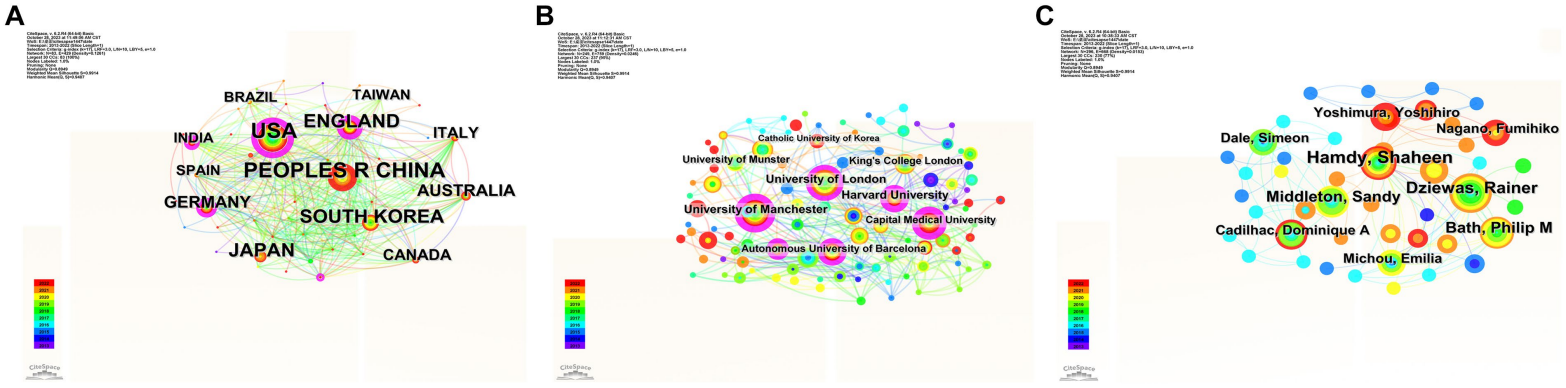
Close cooperation networks among countries **(A)**, institutions **(B)**, and authors **(C)** have been formed globally. Links indicate the presence of co-authorship or co-occurrence relationships.

Figure 3 displays the collaboration maps of countries, institutions, and authors. According to the total link strength, the top three countries were China (*n* = 273), USA (*n* = 251), and England (*n* = 145). The top three institutions were the University of London (*n* = 47), the University of Manchester (*n* = 44), and Harvard University (*n* = 37). As for authors, the top three authors were Hamdy S (*n* = 27), Dziewas R (*n* = 21), and Middleton S (*n* = 16) based on their contributions.

In terms of betweenness centrality, the United States had the highest centrality (*n* = 0.45), and the University of London and the University of Manchester were the top institutions with the highest centrality (*n* = 0.21). The top five authors were Hamdy S (*n* = 0.01), Dziewas R (*n* = 0.01), Middleton S (*n* = 0.01), Bath P (*n* = 0.01), and Michou E (*n* = 0.01).

### Reference analysis

**Figure 4:**
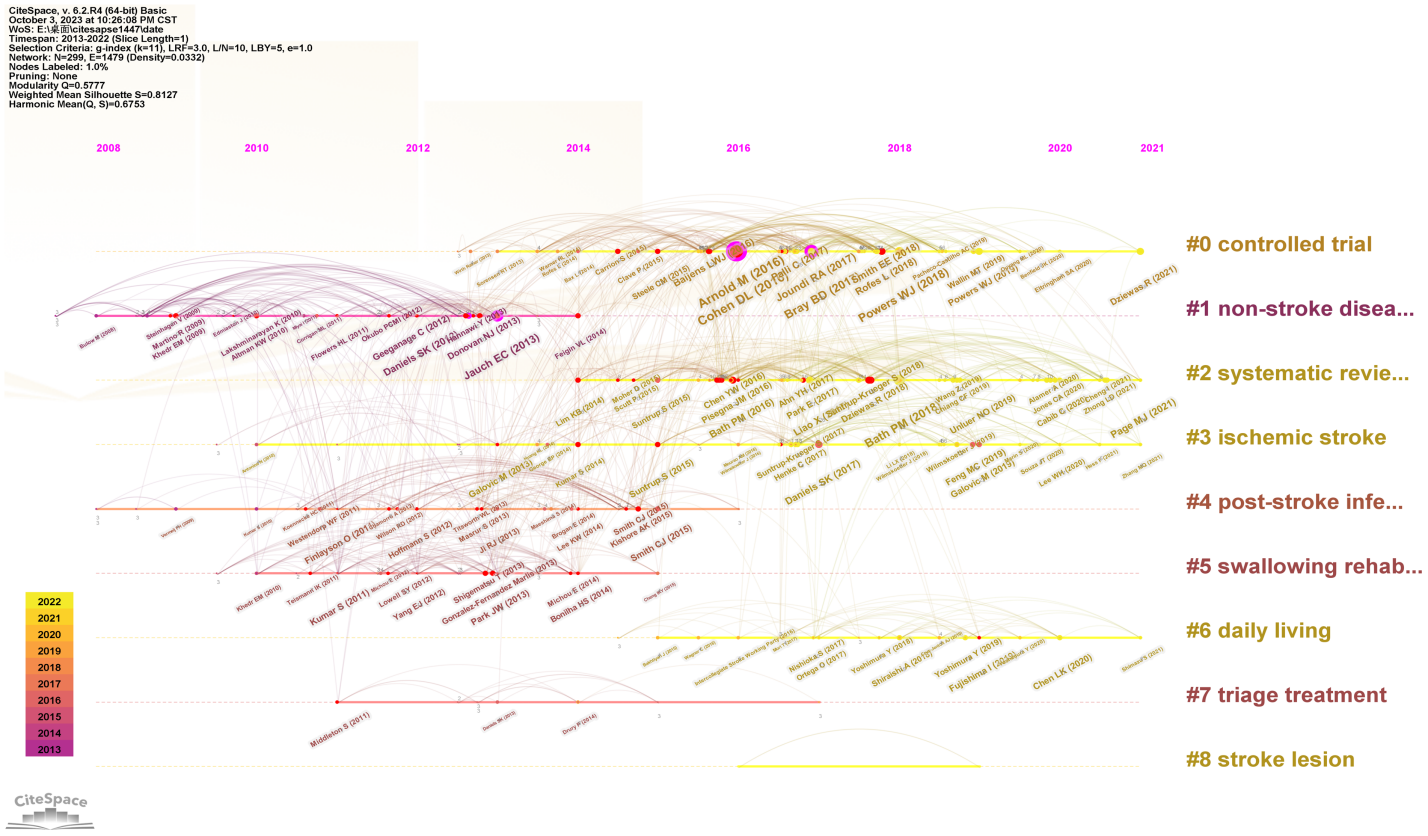
Timeline view of reference co-citation analysis.

Figure 4 presents a timeline view of the references. The reference cocitation analysis grouped the research categories into 26 clusters (#0–26). The largest cluster (#0) consisted of 64 members and was classified as a controlled trial. The most relevant citation to this cluster was “European Stroke Organization and European Society for Swallowing Disorders Guideline for the Diagnosis and Treatment of Post-Stroke Dysphagia” (16). The second-largest cluster (#1), labeled as non-stroke diseases, contained 49 members. The most pertinent citation to this cluster was “Approaches to the Rehabilitation of Dysphagia in Acute Post-Stroke Patients” (29). The third-largest cluster was labeled as ischemic stroke, and the most relevant citation was “Predictors of Complete Oral Feeding Resumption after Feeding Tube Placement in Patients with Stroke and Dysphagia: A Systematic Review” (30).

### Keyword analysis

**Figure 5:**
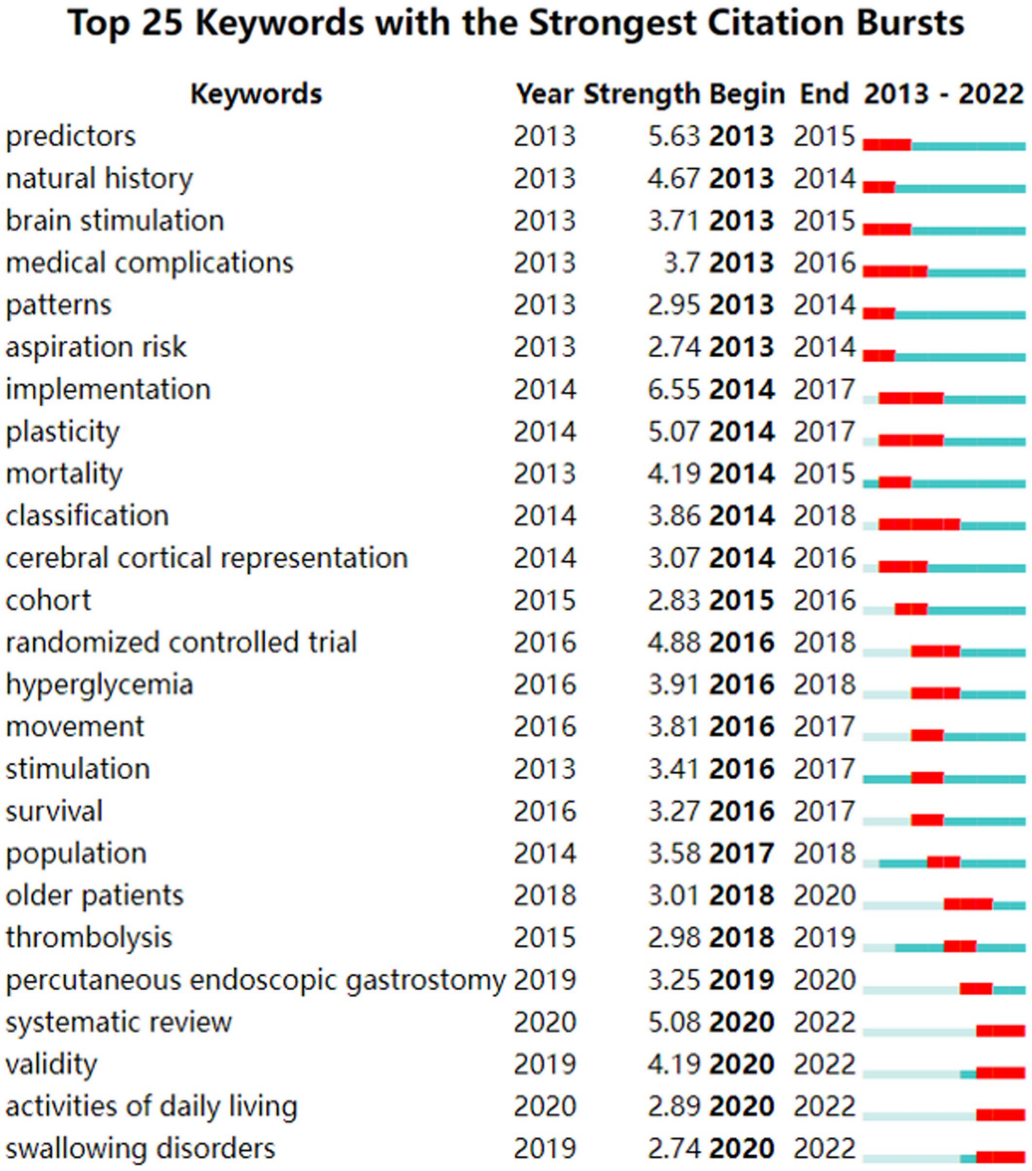
Top 25 keywords with the strongest citation bursts conducted by CiteSpace. The blue bars mean the reference has been published; the red bars mean citation burstness.

Figure 5 displays the top 25 keywords with the strongest citation bursts. The keyword with the highest burst value was “implementation” (*n* = 6.55), followed by “predictors” (*n* = 5.63) and “systematic review” (*n* = 5.08). The keyword “classification” had the longest burst period, lasting from 2014 to 2018. As of the end of 2022, the most frequently cited keywords included “systematic review,” “validity,” “activities of daily living,” and “swallowing disorders.”

## Discussion

### Global research trends of post-stroke dysphagia

This study conducted a systematic and comprehensive bibliometric analysis of research on post-stroke dysphagia in the past 10 years using CiteSpace software. The results showed that the global trend of published papers on post-stroke dysphagia increased during the 10-year period from 2013 to 2022. However, it should be noted that the growth rate of clinical neurology was slower than the overall trend observed in this field. In addition, the global trend in citations increased from 44 to 5,405, with the most significant increase from 2019 to 2022. The increase in clinical neurology was consistent with the overall growth, indicating that dysphagia has received more attention in the field of stroke in recent years, with more and more scholars joining the research, further promoting the development of this field.

From the journal analysis, dysphagia was the Q2 journal with the highest publications and the largest citations. This indicates that publishing high-quality papers on post-stroke dysphagia is a challenge. In addition, among the top 15 journals, Stroke (IF, 2022 = 8.3), the International Journal of Stroke (IF, 2022 = 6.7), and the European Journal of Neurology (IF, 2022 = 5.1) were the Q1 journals with an IF score > 5. This indicates that the articles published in these three journals have a higher academic reference value and are more authoritative. Through analyzing the overlap of dual-maps in published journals to discern the citation trajectories across various fields, it becomes apparent that post-stroke dysphagia is influenced by a range of disciplines extending from medicine, neurology, and clinical health to psychology, education, and sociology. The disciplines mentioned above contribute to addressing this intricate problem from diverse angles, offering distinctive perspectives and approaches. Medicine and neurology concentrate on physiological mechanisms and therapeutic methods, clinical hygiene examines best practices, psychology emphasizes psychological wellbeing, education strives to enhance the disorder through educational means and training; and sociology emphasizes social engagement and support systems (16). Interdisciplinary collaboration and communication are essential for developing comprehensive treatment plans and enhancing patient outcomes in terms of quality of life and prognosis. Future research should take into account the dynamic nature of these field journals.

Among the top 15 authoritative countries ranked by publication quantity, two-thirds were developed countries, while only one-third were developing countries. In addition, although China had the highest number of papers, the United States had the highest number of citations and H-index, and Spain ranked first in citations per paper. This indicates that there are still significant disparities between developed and developing countries in the research on post-stroke dysphagia. This phenomenon may be due to the following reasons: First, some developing countries still focus their research on reducing the mortality rate of stroke, which is the second leading cause of death (31). Second, there are differences in the incidence rate of post-stroke dysphagia between developed countries and developing countries. Meta-analysis shows that the incidence rate in developed countries is approximately 44–61%, while that in developing countries is approximately 37–39% (11). The high incidence rate may prompt developed countries to pay more attention to post-stroke dysphagia. Finally, developed countries have more economic strength, medical investment, talent cultivation, and scientific research environment resources, which affect the output and quality of scientific research results in developed and developing countries. Among the authoritative institutions and authors, the University of London was the top-ranked institution, and Hamdy S was the most influential author. From the perspective of the collaboration network, the United States had the highest centrality. The University of London and the University of Manchester were the top institutions with the highest centrality. These results suggest that countries and institutions with the most publications do not necessarily have the highest degree of betweenness centrality. Future research involving post-stroke dysphagia should strengthen collaboration and cooperation between different countries and institutions to improve research quality.

### Research hotspots and prospects of post-stroke dysphagia

The evolution of a knowledge field can be reflected through keywords. Therefore, keyword analysis can reveal research hotspots and development trends. According to keyword-based counting analysis, dysphagia (*n* = 221) ranked first, followed by aspiration (*n* = 215). Dysphagia is one of the most common complications after stroke (32, 33). Despite multiple advances in the treatment of hyperacute phase and secondary prevention of stroke, the treatment of post-stroke dysphagia remains a neglected research area (33–35). Swallowing and breathing share the pharynx and are both regulated by the medulla oblongata, making them two synchronized complex biomechanical processes (36, 37). During normal swallowing, the airway is closed, and breathing is paused to prevent food from entering the airway (38). Patients with dysphagia may have aspiration due to abnormal airway protection mechanisms, causing food to fall into the respiratory tract (39). Previous studies showed that in stroke patients, the incidence rate of dysphagia was consistent with pneumonia (11, 32). However, conventional diagnostic methods for dysphagia have limited accuracy in predicting aspiration and respiratory disease (40). In addition, there was insufficient randomized controlled trial data to determine the impact of screening programs for dysphagia on reducing post-stroke pneumonia (41). Therefore, more research is needed to compare the effectiveness of different screening methods for dysphagia in the future, and incorporating measurable objective assessments into clinical diagnosis is necessary. This may be the key to developing new treatment strategies. The keyword “classification” had the longest burst period. Warnecke et al. found that based on the flexible endoscopic evaluation of swallowing (FEES), the neurogenic dysphagia phenotype can be divided into seven categories (42). Stroke commonly manifests as “premature bolus spillage,” “delayed swallowing reflex,” “residual material in the piriform sinus,” and “pharyngolaryngeal movement disorder” (12). Parkinson’s disease often appears as “residual material in the valleculae” and “pharyngolaryngeal movement disorder” (43). Myasthenia gravis is commonly characterized by “fatigue-prone muscle weakness” (44). “Complex disorder” with a heterogeneous dysphagia pattern is more prevalent in amyotrophic lateral sclerosis (45). Therefore, the dysphagia phenotype is beneficial for the differential diagnosis of post-stroke dysphagia.

The subject category analysis showed that clinical neurology, neurosciences, and rehabilitation were the main categories of research on post-stroke dysphagia, indicating that research on post-stroke dysphagia from the perspective of clinical neurology focuses on rehabilitation. In the reference analysis, the most relevant citer to the cluster “swallowing rehabilitation “was “Transcranial non-invasive brain stimulation in swallowing rehabilitation following stroke--a review of the literature” (46). This study found that, based on available evidence, non-invasive brain stimulation may provide a useful adjunctive therapy for post-stroke dysphagia rehabilitation. In addition, according to the timeline perspective of literature analysis, “daily living” has also been a hot topic in recent years. Dysphagia may prevent individuals from living independently and returning to work due to conditions such as being unable to eat orally, using nasal feeding tubes, developing aspiration pneumonia, experiencing malnutrition, and suffering from mental disorders, which significantly impact the quality of life (47). Therefore, more high-quality research on non-invasive interventions for post-stroke swallowing dysfunction is needed in the future to improve the quality of life of patients with post-stroke dysphagia.

Although this study reveals the trends and hotspots of post-stroke dysphagia, there are still some topics and areas that have not been fully studied. For example, the efficacy of swallowing exercises and rehabilitation programs, the specific needs of elderly patients, and the application of technology in diagnosis and management (39, 48–50). In-depth research on these topics will help medical practitioners better understand post-stroke dysphagia and provide more effective treatment and management strategies.

### Strengths and limitations

As far as we know, this study represents the first bibliometric and visual analysis of post-stroke dysphagia, drawing from literature published between 2013 and 2022. In addition, this study reviews the progress and trends of research on post-stroke dysphagia worldwide. However, it should be noted that there are several limitations. First, considering the limitations of CiteSpace software and in order to ensure the quality of the retrieved publications and the integrity of the information, we only searched the core collection of Web of Science, which may have omitted important literature from other databases. Second, in the search strategy, we did not incorporate all keywords associated with dysphagia. To achieve a more comprehensive understanding of this field, future research can refine the search strategy based on this foundation, leading to more robust evidence support. Finally, our study only included publications from 2013 to 2022 and did not incorporate the most recently published high-quality papers. This may lead to an incomplete representation of the latest research dynamics and advancements, particularly in rapidly evolving fields such as post-stroke dysphagia.

## Conclusion

In conclusion, this study may help researchers reveal the publication patterns and emerging trends of post-stroke dysphagia from 2013 to 2022. The most influential authors, institutions, journals, and countries were Hamdy S, University of London, Dysphagia, and the People's Republic of China. The visual map displays the hot research directions of post-stroke dysphagia studies in recent years, including dysphagia and aspiration, dysphagia classification, dysphagia rehabilitation, and daily living. In stroke patients with dysphagia, accurate identification of the type of swallowing disorder and in-depth exploration of novel rehabilitation techniques, such as non-invasive brain stimulation, contribute to the development of more scientific and effective treatment protocols. Furthermore, precise assessment of the risk of aspiration and the implementation of appropriate nursing interventions contribute to reducing the risk of pneumonia and, ultimately, significantly enhancing the patients’ quality of life. Therefore, it is crucial to conduct large-sample randomized controlled trials on screening programs and non-invasive intervention methods for post-stroke dysphagia in the future.

## Data availability statement

The original contributions presented in the study are included in the article/Supplementary material, further inquiries can be directed to the corresponding authors.

## Author contributions

BG: Data curation, Writing – original draft. ML: Writing – review & editing. ZW: Conceptualization, Writing – review & editing. ZY: Conceptualization, Writing – review & editing.
