# Supplementary material for: Research hotspots and frontiers in post-stroke dysphagia: a bibliometric analysis study
Source: Front Neurol. 2024 Feb 2;15:1343469. doi: 10.3389/fneur.2024.1343469 (PMC10869480; doi:10.3389/fneur.2024.1343469)
Supplement: Supplementary file 1 [file Data_Sheet_1.pdf]

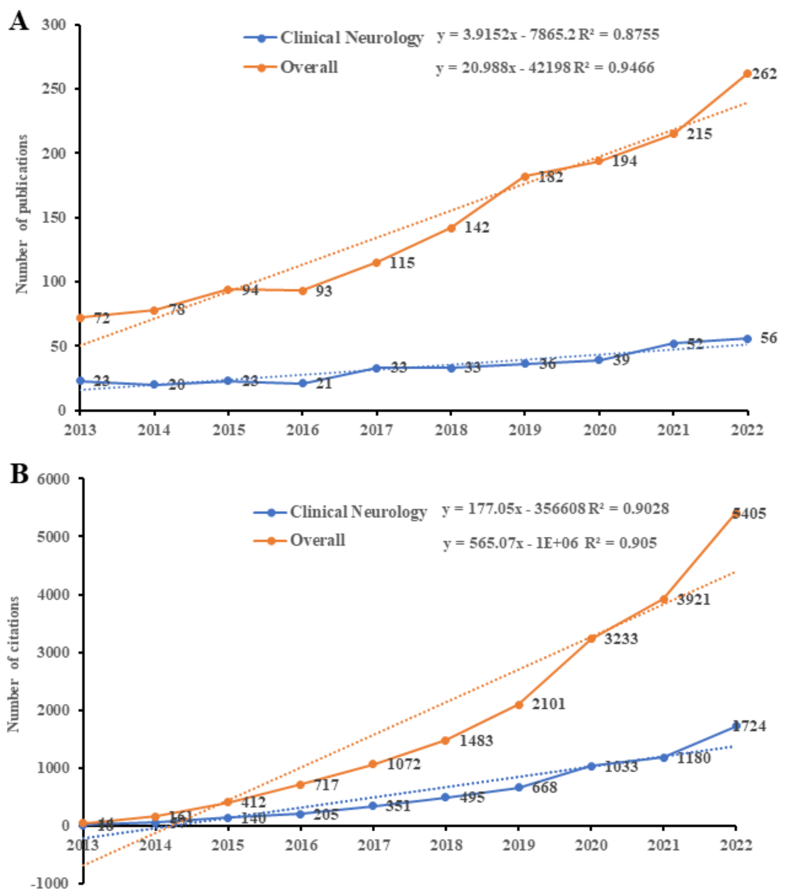

Figure 1. The distribution of publications and citations. Orange represents the overall trend in the broader field, and blue represents the trend in Clinical Neurology. (A) The distribution of annual publications of post-stroke dysphagia from 2013 to 2022. (B) The distribution of annual citations of post-stroke dysphagia from 2013 to 2022.

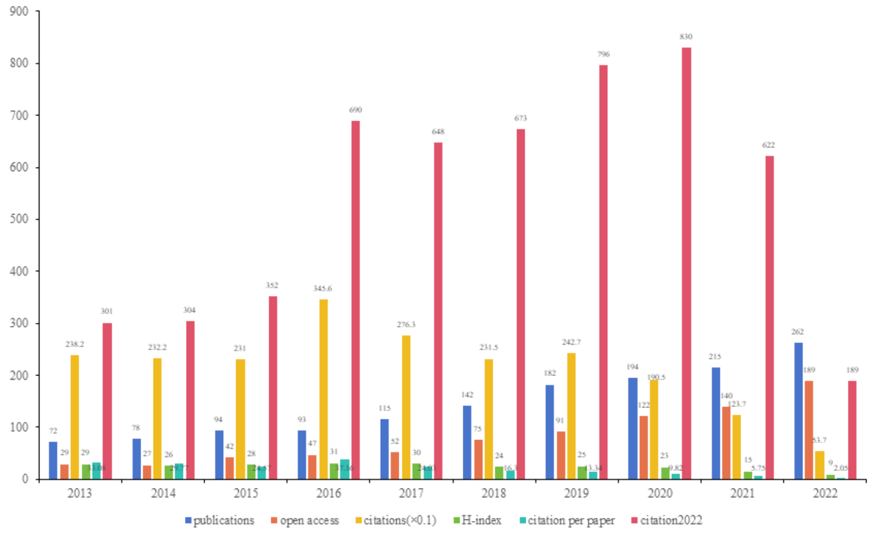

Figure 2. The number of open-access articles, publications, citations（×0.1）, citations per paper, H-index, and citations in 2022 for per year.

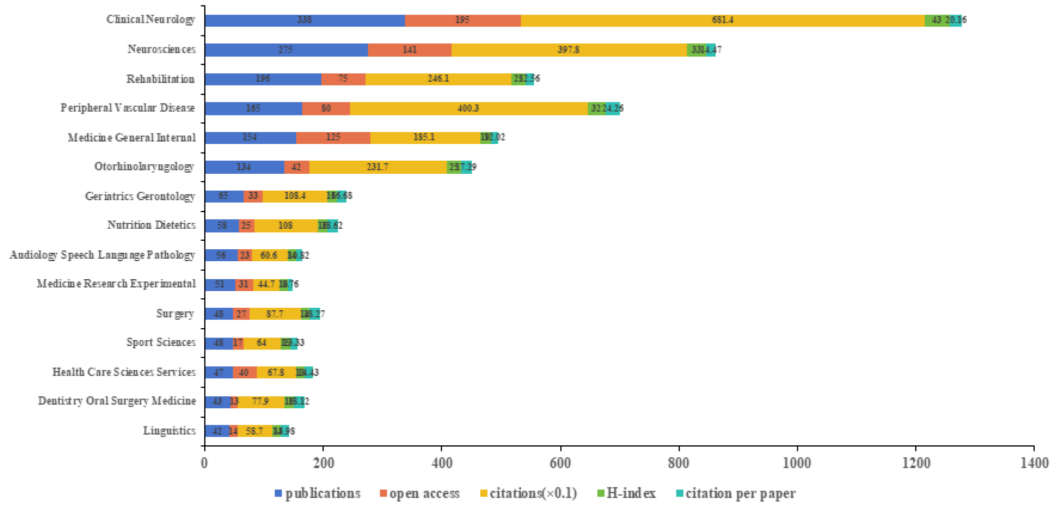

Figure 3. Top 15 subject categories of Web of Science in terms of open-access articles, publications, citations（×0.1）, citations per paper, and H-index.

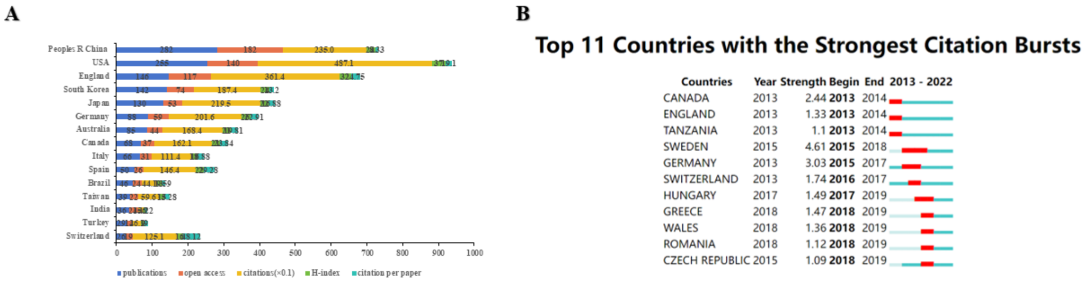

Figure 4. Countries contributed to the publication of research on post-stroke dysphagia. (A) Top 15 countries in terms of open-access articles, publications, citations（×0.1）, citations per paper, and H-index. (B) Top 11 countries with the strongest citation bursts conducted by CiteSpace. The Blue bars mean the reference had been published; the red bars mean citation burstness.

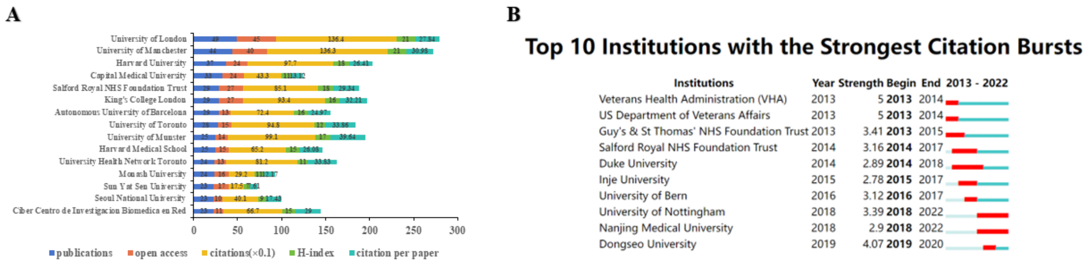

Figure 5. Institutions contributed to the publication of research on post-stroke dysphagia. (A) Top 15 institutions in terms of open-access articles, publications, citations（×0.1）, citations per paper, and H-index. (B) Top 10 institutions with the strongest citation bursts conducted by CiteSpace. The Blue bars mean the reference had been published; the red bars mean citation burstness.

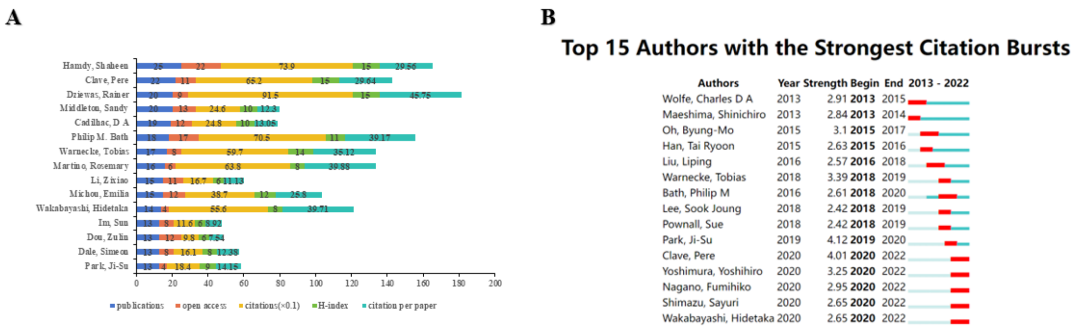

Figure 6. Authors contributed to the publication of research on post-stroke dysphagia. (A) Top 15 authors in terms of open-access articles, publications, citations（×0.1）, citations per paper, and H-index. (B) Top 15 authors with the strongest citation bursts conducted by CiteSpace. The Blue bars mean the reference had been published; the red bars mean citation burstness.
